# Alcohol Use Disorder Pharmacotherapy in Patients With Alcohol-Related Liver Disease: A Scoping Review

**DOI:** 10.1155/cjgh/6455092

**Published:** 2025-04-03

**Authors:** Manisha Jogendran, Louis Huynh, Jennifer A. Flemming

**Affiliations:** ^1^Department of Medicine, Queen's University, Kingston, Ontario, Canada; ^2^Department of Public Health Sciences, Queen's University, Kingston, Ontario, Canada

## Abstract

**Introduction:** Alcohol-associated liver disease (ALD) is one of the most common causes of cirrhosis. Pharmacotherapy for alcohol use disorder (AUD) can improve abstinence rates in patients with cirrhosis, however, there is limited data on how these therapies affect liver-related outcomes.

**Methods:** A scoping review was completed using multiple electronic search databases. Articles exploring pharmacotherapy for AUD and outcomes for ALD were included. The primary outcome of this study was liver outcomes after receiving pharmacotherapy for AUD, including decompensated cirrhosis, mortality, progression of ALD, and need for liver transplantation.

**Results:** A total of 2521 studies were screened and 3 were selected. A total of 45,948 patients were included, 43,863 (98%) of patients were male, and the mean age was 58.7. Only 2299 (5%) of patients received AUD pharmacotherapy. Receipt of AUD pharmacotherapy was found to be associated with decreased hepatic decompensation and mortality in 2 out of 3 studies.

**Conclusion:** There are limited studies that explore AUD pharmacotherapy and ALD outcomes. Medications AUD may improve hepatic outcomes; however, further prospective studies need to be completed to explore this association.

## 1. Introduction

It is estimated that 25% of deaths from cirrhosis across the world are related to alcohol use, and this may worsen as global alcohol consumption is projected to increase. In North America, alcohol-associated liver disease (ALD) is the second most common cause of cirrhosis and is among the leading causes for liver transplantation [1–4]. Abstinence from alcohol consumption is the most effective method of preventing disease progression, and has been shown to reverse hepatic steatosis, prevent hepatic decompensation, and improve survival even in patients with advanced disease [5–7]. Conversely, continued alcohol consumption is associated with progression of liver disease to cirrhosis and hepatocellular carcinoma [8, 9].

The pathogenesis of alcohol use disorder (AUD) has been linked to neurotransmitter pathways involved in motivation and reward, particularly the mesolimbic dopamine system. Alcohol stimulates the release of dopamine from the ventral tegmental area into the nucleus accumbens and prefrontal cortex, resulting in euphoria and thereby reinforcing drinking behaviour. With prolonged use, neuroplasticity occurs of this system occurs and results in addiction. Numerous other neurotransmitters mediate the effect of alcohol on dopamine, including GABA, glutamate, corticotropin-releasing factor, 5-HT, and endogenous opioids [10]. These neurotransmitters are also the target of various pharmacotherapies used to treat AUD. Furthermore, alcohol use can disturb the balance between GABA, the primary inhibitory neurotransmitter, and glutamate, the major excitatory neurotransmitter. With long term use, the brain attempts to compensate to restore this balance which manifests as increased tolerance to alcohol's sedative effects. Conversely, abrupt cessation after chronic alcohol use results in an unopposed activity of the excitatory system, resulting in ongoing dependence for alcohol use to avoid alcohol withdrawal [11].

A combination of medical and behavioral therapy is effective in promoting abstinence in AUD in patients with ALD [12]. There are currently three FDA-approved medications (disulfiram, naltrexone, and acamprosate) that have been associated with increased abstinence [13]. Additional agents that have not been FDA-approved include gabapentin, topiramate, and baclofen. Pharmacotherapy aims to target pathways that reduce alcohol consumption [14]. Naltrexone acts as a nonselective antagonist of *μ*, *κ*, and *δ*-opioid receptors, thereby blocking the endogenous opioids that mediate the dopaminergic reward system targeted by alcohol. Acamprosate is thought to stabilize the imbalance between excitatory and inhibitory neurotransmitters, possibly by antagonizing NMDA receptors and agonizing GABA receptors [15]. Disulfiram inhibits aldehyde dehydrogenase 2, increasing acetaldehyde levels and causing adverse effects that promote alcohol avoidance. Gabapentin has a similar GABA-related mechanism, though it remains unclear. Baclofen is a GABA-B receptor agonist that helps restore chronically depleted GABA levels in AUD. Topiramate facilitates GABA-A receptors, which reduces dopamine levels in the mesocorticolimbic system and decreases alcohol intake [16].

Despite the proven efficacy of pharmacotherapy in treating AUD, medications for AUD remain underutilized in patients with less than 10% of patients receiving pharmacotherapy [17]. While it is known that medications for AUD improve abstinence rates in patients with ALD, the literature on whether these therapies affect liver-related outcomes remains limited [12]. Therefore, we completed a scoping review to assess the association between AUD pharmacotherapy and complications of ALD.

## 2. Materials and Methods

### 2.1. Search Strategy

A scoping review was completed using the PRISMA extension for scoping reviews [18]. An electronic search was completed using EMBASE (1974–present), MEDLINE (1946–present), EBM Reviews–Cochrane Central Register of Controlled Trials (1946–present) and EBM Reviews–Cochrane Database of Systematic Reviews (2005–present). The medical subject terms searched were “alcohol liver disease” OR “liver cirrhosis” AND “alcohol use” OR “alcohol use disorder” AND “pharmacotherapy” OR “drug treatment.” From the articles selected, the references were screened to determine if additional articles met the criteria.

### 2.2. Study Selection

Manisha Jogendran and Louis Huynh conducted the initial screening by individually assessing the titles and abstracts of studies to identify full-text articles exploring pharmacotherapy for AUD and impacts on patients with ALD. Articles that met inclusion criteria underwent a comprehensive review.

### 2.3. Data Extraction

Manisha Jogendran and Louis Huynh, conducted the extraction and tabulation of the following data: (a) Study characteristics encompassing title, author, location, study type, region, and study period; (b) demographic details covering sex and age; (c) liver disease factors including etiology and severity of liver disease; (d) substance use history including severity of AUD, concomitant substance use disorder; (e) other psychiatric comorbidities. The primary outcome of this study is liver outcomes after receiving pharmacotherapy for AUD, including decompensated cirrhosis, mortality, progression of ALD, and need for liver transplantation. Studies were stratified based on no pharmacological treatment (includes behavioural therapy only) vs AUD treatment (includes pharmacotherapy and behavioral therapy combined and pharmacotherapy alone).

## 3. Results

**Figure 1:**
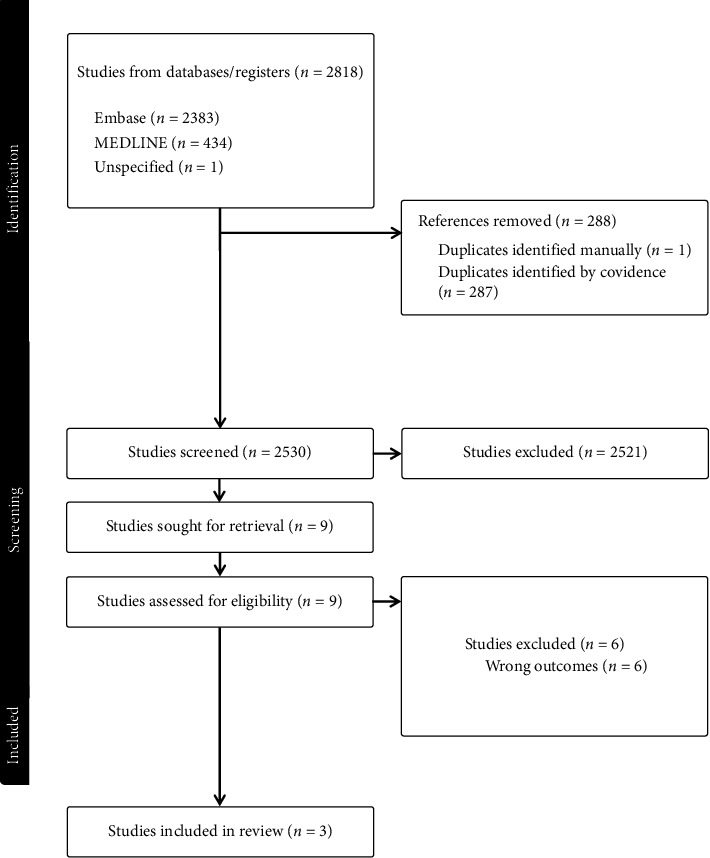
Search strategy.

A total of 2521 studies were screened during the search, from which 3 were selected for inclusion [19–21]. See Figure 1 for full details. All 3 selected studies were retrospective cohort studies and conducted in North America. A total of 45,948 patients were included. A total of 43,649 (95%) received no AUD treatment while 2299 (5%) received pharmacological treatment. Of those who received treatment, 1831 (80%) received pharmacotherapy only and 468 (20%) received both therapies. In terms of AUD pharmacotherapy, all studies explored acamprosate and naltrexone, 2/3 studies explored disulfiram and 1/3 studies explored gabapentin, topiramate, baclofen.

### 3.1. Demographics

Demographic data were available in 2/3 studies. Among those who received any treatment, the mean age was 54.1 years vs. 58.9 years in those without AUD treatment. The majority of patients were male, 42,404/43,300 (98%) were male in the nonpharmacotherapy group, and 1459/1513 (96%) in the pharmacotherapy group. See Table 1 for full details.

### 3.2. Liver Disease

2 out of 3 studies had hepatitis C (HCV) status available. A concomitant diagnosis of HCV was present in 20,125/43,300 (46%) of patients in the nonpharmacotherapy group vs 471/1513 (31%) in the pharmacotherapy group. MELD-Na and AUDIT-C scores were reported in 2 out of the 3 studies. Among patients who had MELD-Na scores available, the average score was 10.9 in the nonpharmacotherapy group and 10.4 in the pharmacotherapy group. Among patients with AUDIT-C scores available, the average AUDIT-C was 5.06 in the nonpharmacotherapy group and 9.12 in the pharmacotherapy group.

### 3.3. Psychiatric Comorbidities

2 out of 3 studies reported data on psychiatric comorbidities and substance use disorders. Psychiatric comorbidities were present in 18,043/43,300 (42%) in the nonpharmacotherapy group and 902/1513 (60%) in the pharmacotherapy group. Concomitant substance use disorders (e.g., nicotine, opioids, cocaine, cannabis, or other drugs) were present in 20,078/43,300 (46%) in the nonpharmacotherapy and 1076/1513 (71%) in the pharmacotherapy group.

### 3.4. Hepatic Decompensation

Hepatic decompensation was the outcome in 2 out of 3 studies. Rogal et al. looked at hepatic decompensation at 180 days follow up from their index AUD diagnosis. They defined hepatic decompensation as variceal bleeding, hepatic encephalopathy, hepatopulmonary syndrome, ascites, spontaneous bacterial peritonitis, or hepatorenal syndrome based on ICD-9-CM codes. New hepatic decompensation occurred in 3518/35,055 (10%) who received no AUD therapy and pharmacotherapy 36/627 (6%). Receipt of any type of AUD therapy was associated with a significantly decreased odds of hepatic decompensation (AOR = 0.63).

Details of how hepatic decompensation was defined by Vannier et al. were not outlined. Patients were followed for a mean duration of 7.8 years in the treated group and 8.6 years in the untreated group. In their study, receiving pharmacotherapy for AUD was associated with a reduced incidence of hepatic decompensation (AOR = 0.35), in addition to a longer time to first hepatic decompensation after cirrhosis diagnosis (6.3 vs. 2 years). The association between pharmacotherapy and reduced hepatic decompensation persisted in 105 patients who were initiated on pharmacotherapy only after a diagnosis of cirrhosis was established (AOR = 0.41).

### 3.5. Death

Death was reported as an outcome in 2 out of 3 studies. Rabbie et al. found that use of pharmacotherapy for AUD in the first year after cirrhosis diagnosis was associated with a 20% reduced hazard of all-cause mortality. Furthermore, longer duration of exposure to pharmacotherapy was associated with improved survival. There was no difference in mortality between patients on acamprosate versus naltrexone. Similarly, Rogal et al. found that any therapy for AUD (i.e., pharmacotherapy and/or behavioural therapy) was associated with decreased mortality a 180-day-follow up (adjust odds ratio = 0.87). At 180-day follow-up, death occurred in 1327/35,055 (3.8%) who received no AUD treatment and 9/627 (1.4%) who received pharmacotherapy.

In our review of the literature, we did not find any studies that look at transplant or liver disease progression as an outcome. None of the included studies reported data on adverse events.

## 4. Discussion

The incidence of liver disease related to alcohol use is rising in our population, and it is predicted to continue increasing [3]. There are very limited studies studying the association of pharmacotherapy and ALD outcomes. The results of our scoping review suggest that patients with ALD who are treated with pharmacotherapy or behavioral therapy for AUD have decreased rates of hepatic decompensation events and less death. However, there are no outcomes studying transplant rates in these patients and progression of alcohol liver disease.

Among patients with ALD, those who received pharmacotherapy alone had higher rates of psychiatric comorbidities and concomitant substance use disorder compared to patients who received no therapy. Similar trends are seen in patients with AUD but no liver disease [22, 23]. The findings could reflect the fact that patients with liver disease and concomitant psychiatric comorbidities are more likely to seek care from providers that often prescribe pharmacotherapy for AUD, as receiving mental health care services is associated with increased receipt of pharmacotherapy for AUD [24]. Furthermore, patients who received pharmacotherapy had higher AUDIT-C scores, which may reflect more severe disease warranting treatment. Finally, genetic and environmental factors that predispose individuals to develop AUD also play a role in the pathogenesis of other psychiatric and substance use disorders, explaining the high rates of comorbidity seen in this patient population [25].

In our review, approximately 45% of patients with ALD also had a diagnosis of HCV. The presence of HCV in patients with AUD is thought to be higher than the general population, with an estimated prevalence ranging from 2.1% to 51% [26]. Injection drug use and the presence of ALD have been shown to be associated with a higher risk of HCV in patients with AUD. For example, one large systemic review found the prevalence of HCV to be 32.9% in those with severe liver disease [27]. Interestingly, while we found that patients treated with pharmacotherapy had higher rates of comorbid substance use disorder, they actually had lower rates of HCV (31% vs. 46%). This could be explained the fact that, while substance use in general is captured in our data, none of the studies we found looked specifically at injection drug use which would be the primary risk factor for HCV. On the other hand, it is also possible that patients who were more likely to be treated for their AUD with pharmacotherapy may have also been more likely to receive antiviral treatment of their HCV, as these two disorders are often treated concurrently at integrated care centres [28]. This intricate association between AUD and HCV, in addition to other substance use and psychiatric disorders, underscores the need for taking a holistic and multidisciplinary approach in treating patients with ALD and preventing the downstream consequences of hepatic decompensation.

Importantly, our data suggests that pharmacotherapy is associated with reduced hepatic decompensation and mortality. Despite this, less than 10% of patients in our studies received pharmacotherapy for AUD. Similarly, a large cohort study of over 60,000 patients with ALD in the United States demonstrated that only 10% received substance use or mental health services and less than 1% received FDA-approved pharmacotherapy for AUD [29]. This may be due to several barriers associated with receiving AUD treatment among patients with ALD. These include social determinants of health preventing patients from seeking AUD care, stigma surrounding AUD, lack of clinician experience in treating AUD in patients with concomitant ALD, and lack of resources [30–32]. Given the persistent underutilization of pharmacotherapy for AUD in ALD, along with increasing evidence demonstrating its efficacy in improving liver-related outcomes, it is crucial to address these barriers to AUD treatment in this patient population.

Our review of the literature did not reveal any studies assessing liver transplantation as a primary outcome. While liver transplantation remains the only curative therapy in patients with end-stage ALD, many patients with ALD often are not eligible for transplant candidacy due to concerns about ongoing high-risk alcohol use, and a significant number of patients die during transplant evaluation [33, 34]. Understanding whether pharmacotherapy can reduce the necessity for liver transplantation would provide invaluable information on improving the mortality and prognosis of patients with ALD.

Furthermore, the studies included in our scoping review showed a limited focus on women, despite a recent rise in the epidemiology of AUD among this group [35]. Biological changes have contributed to increased alcohol-related issues and higher consumption. Women also face social barriers, such as stigma and childcare responsibilities, which impact their retention in treatment programs [36]. Additionally, research often overlooks gender-specific approaches in pharmacotherapy and cognitive-behavioral therapy, despite significant social and biological differences that warrant such focus [37].

Although our study had many strengths, there are also limitations. Firstly, we only included 3 studies, which were heterogeneous in the study design and only included retrospective studies. There were also study groups which included both behavioral therapy and pharmacological therapy which makes it difficult to classify the data to be attributed solely to pharmacological therapy. Unfortunately, none of these studies explored transplant outcomes and progression of liver disease, both important factors to explore in patients with ALD. Furthermore, none of the included studies reported adverse event outcomes. Little work has been done studying the adverse effects of medications for AUD in cirrhosis, despite potential theoretical risks associated with their use in liver disease. Naltrexone has previously been associated with hepatotoxicity, although recent retrospective data demonstrated that its use was not associated with the development of drug-induced liver injury in patients with cirrhosis [38, 39]. Acamprosate is contraindicated in patients with renal dysfunction, limiting its use in the setting of decompensated cirrhosis [40]. Future prospective studies on medications for AUD in patients with cirrhosis should report safety outcomes, as these have remained largely understudied to date.

In conclusion, our scoping review demonstrates there are limited studies exploring the association between AUD pharmacotherapy and ALD. AUD pharmacotherapy may benefit and improve hepatic outcomes in patients with ALD. Despite the significance of pharmacotherapy in treating AUD there remains a substantial gap in research within this area. Moving forward, further prospective studies need to be completed studying this association.

## Figures and Tables

**Table 1:** Study characteristics and outcomes.

Study	Country	Treatment group	Patients (*N*)	Age (years)	Male sex, *n* (%)	Concomitant hepatitis C, *n* (%)	Severity of liver disease (MELD-Na)	Severity of AUD (AUDIT)	Psychiatric disorder, *n* (%)	Current nicotine use, *n* (%)	Substance use disorder, *n* (%)	Death, *n* (%) or OR	Decompensation, *n* (%) or OR
Rabie et al., 2023	USA	No treatment	8245	59	8061 (98%)	2672 (32%)	11.9	10.5	3219 (39%)	3842 (47%)	2709 (33%)		Unknown
		Treatment	886	54	851 (96%)	225 (25%)	10.9	11.0	536 (60%)	396 (45%)	394 (44%)	0.80	Unknown

Rogal et al., 2020	USA	No treatment	30,594	59.3	30,025 (98%)	14,862 (49%)	10.7	3.6	12,313 (40%)	6742 (22%)	3931 (13%)	1203 (4%)	3267 (12%)
		Behavioural only	4461	55.7	4318 (98%)	2591 (58%)	10.3	5.0	2511 (56%)	1069 (24%)	1785 (40%)	124 (3%)	251 (7%)
		Pharmacological only	159	54.8	155 (98%)	53 (33%)	9.8	6.0	124 (78%)	41 (26%)	(22%)	1 (1%)	8 (6%)
		Both	468	53.9	453 (97%)	193 (41%)	9.7	6.6	242 (52%)	96 (21%)	114 (24%)	8 (2%)	28 (7%)

Vannier et al., 2022	USA	No treatment	349	Unknown	Unknown	Unknown	Unknown	Unknown	Unknown	Unknown	Unknown	Unknown	
		Treatment	786	Unknown	Unknown	Unknown	Unknown	Unknown	Unknown	Unknown	Unknown	Unknown	0.35

## Data Availability

Data sharing not applicable to this article as no datasets were generated or analysed during the current study.
